# Stable Isotopes for the Study of Energy Nutrient Metabolic Pathways in Relation to Health and Disease

**DOI:** 10.3390/metabo16040231

**Published:** 2026-03-31

**Authors:** Dalila Azzout-Marniche, Daniel Tomé

**Affiliations:** 1 INRAE, UMR PNCA, AgroParisTech, Université Paris-Saclay, 91120 Palaiseau, France; dalila.azzout-marniche@agroparistech.fr; 2 AgroParisTech, Université Paris-Saclay, 91120 Palaiseau, France

**Keywords:** stable isotopes, nutrient metabolism, glucose, fatty acid, amino acid, protein

## Abstract

**Background:** Stable isotope-based analytical methods have brought about a significant transformation in the study of energy nutrient metabolism, enabling precise in vivo measurement of metabolic fluxes at systemic, tissue, and organ-specific levels in both healthy and diseased states. The regulation of these metabolic fluxes is governed by dynamic interactions between proteins, lipids, carbohydrates, and their precursors—such as glucose, fatty acids, and amino acids—as well as final metabolic products. **Discussion:** Advanced analytical technologies, including nuclear magnetic resonance (NMR) spectroscopy and mass spectrometry (MS), which can offer enhanced precision, have been developed for investigating nutrient metabolism and fluxes in humans, providing precise information on metabolic pathways. These techniques have primarily utilized stable isotopes, such as ^2^H, ^13^C, ^15^N, and ^18^O, which have largely replaced radioactive isotopes and are now central to metabolic research. These isotopes have been used to label glucose, fatty acids, or amino acids—the main biomolecular precursors—enabling detailed investigation at systemic, tissue, and organ-specific levels of carbohydrate, lipid, and protein metabolism, and revealing pathway alterations associated with diseases conditions, such as diabetes, non-alcoholic fatty liver disease, cardiovascular disorders, and cancer. The use of deuterium oxide (D_2_O) has allowed for long-term metabolic studies, providing a cost-effective and less invasive means to monitor metabolic changes over days to months. Total daily energy expenditure can be measured in free living conditions by the doubly stable isotopes ^2^H- and ^18^O-labeled water method. Stable isotope tracing, combined with advanced imaging and modeling, has also been instrumental in assessing body composition, energy expenditure, and nutrient bioavailability. Collectively, these methods have expanded our understanding of human physiology and disease, supporting the development of novel diagnostic tools, the identification of new biomarkers, and the tailoring of nutritional and therapeutic interventions. **Conclusions:** This review aimed to provide an overview of the applications of stable isotopes for the study of energy nutrient metabolic pathways. The ongoing integration of stable isotope approaches with artificial intelligence, omics technologies, and miniaturized detection techniques could promise to further refine our understanding of human metabolism and drive advances in personalized medicine.

## 1. Introduction

Stable isotope-based methods are utilized to study and measure in vivo energy nutrient metabolic fluxes at systemic, tissue, and organ-specific levels in both healthy and disease conditions [1]. Metabolic fluxes of nutrients result from continual regulation of biological processes including nutrient intake, synthesis, interconversion, catabolism, and excretion. In particular, the homeostasis of energy nutrients depends on the dynamic interactions within complex networks of proteins, lipids, and carbohydrate biomolecules, along with their precursors—such as glucose, amino acids, and fatty acids—and associated low-molecular-weight final metabolic end-products. To elucidate and characterize these intricate pathways, advanced analytical technologies with enhanced precision and/or sensitivity have been developed, including nuclear magnetic resonance (NMR) spectroscopy and mass spectrometry (MS) [2,3]. These techniques primarily utilize stable isotopes, which have substantially replaced radioactive isotopes for investigations of nutrient metabolism and fluxes in humans [2] (Table 1).

In addition to the most common isotopes—such as ^1^H, ^12^C, ^14^N, and ^16^O—there are stable isotopes including ^2^H, ^13^C, ^15^N, and ^18^O that have additional neutrons. These stable isotopes are used to label biomolecule precursors; for example, ^2^H- or ^13^C-labeled glucose tracers are utilized to study carbohydrate metabolism, ^2^H- or ^13^C-labeled fatty acid tracers to investigate lipid metabolism, and ^2^H-, ^13^C-, ^15^N-, or ^18^O-labeled amino acids tracers to examine protein metabolism. As a main approach, stable isotope-labeled molecules are administered (ingested or infused) and their metabolism monitored by targeted analysis of such isotopes at different levels from samples collected in the body and in urine and breath. Generally, except for certain metabolic pathways, stable isotope-labeled precursors and metabolites behave similarly to their most common unlabeled isotopes forms, and analyzing the fate of stable isotope-labeled compounds can provide insight into the behavior of their natural unlabeled counterparts.

Deuterium oxide (D_2_O or ^2^H_2_O, heavy water) serves as a versatile stable isotope tracer in metabolic research, allowing for the simultaneous labeling of multiple substrates [4,5,6]. When ingested, D_2_O rapidly distributes throughout the body’s water stores—within approximately two hours—and integrates into metabolic pathways that use water as an intermediate. This process results in the labeling of key biological precursors, such as glucose, fatty acids, and amino acids, which are essential for synthesizing of several other biomolecules, and traces carbohydrate, lipid, and protein metabolism. Thanks to its relatively long half-life in body water (around 9 to 11 days), D_2_O enables metabolic measurements to be obtained over extended periods, from several days to weeks or even months, with either a single dose or regular daily or weekly administration. Moreover, total daily energy expenditure is measured by the doubly stable isotopes ^2^H- and ^18^O-labeled water method, which also enables measurements in free living conditions to be made over extended periods [1,7,8].

This review illustrates the use of stable isotopes to study the metabolism of energy nutrients and provides examples intended to highlight the novelty of the applications of stable isotopes. Nuclear approaches, particularly stable isotope tracing methods that are sometimes combined with imaging techniques, can play a decisive role by complementing conventional biochemical methods, allowing the assessment of numerous metabolic parameters with a high degree of precision and safety [9,10,11,12]. Nuclear and imaging techniques can also be used to determine body composition between fat and lean mass, to measure energy expenditure, and to assess the bioavailability, metabolism, and storage of various nutrients essential for physiological functions [13,14,15,16].

## 2. Monitoring Glucose Metabolism

**Figure 1:**
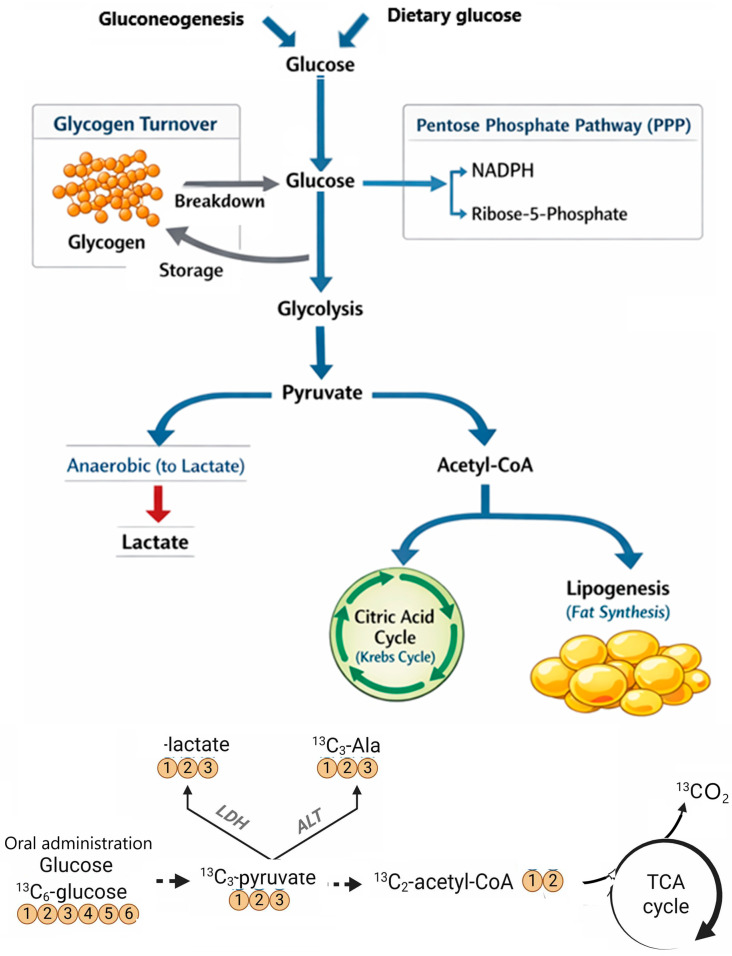
Glucose and ^13^C_6_-glucose metabolic pathways. Lactate dehydrogenase (LDH); alanine aminotransferase (ALT).

Glucose serves as the principal energy source for cellular processes, supplying ATP and carbon substrates that are essential for cellular maintenance, growth, and proliferation. It is a critical energy substrate for specific tissues—such as the brain, erythrocytes, and fast-twitch skeletal muscle—while also acting as an energy source in conjunction with fatty acids and amino acids in other organs like the heart when glucose is less available, such as in fasting state. The metabolism of glucose involves multiple pathways and metabolic routes of carbon flux, including glycolysis, the pentose phosphate pathway (PPP), glycogen metabolism, the tricarboxylic acid (TCA) cycle, and fatty acid and amino acid metabolism (Figure 1). During glycolysis, glucose is converted to pyruvate, which can subsequently be metabolized to lactate or transformed into acetyl-CoA via pyruvate dehydrogenase. Acetyl-CoA may then enter the citric acid cycle or function as a precursor for lipogenesis through citrate synthesis. Additionally, glucose may be directed into the pentose phosphate pathway for nucleic acid biosynthesis or stored as glycogen [17]. The metabolic fate of glucose can be assessed using multiple glucose tracers [12] (Table 2). The expression of enzymatic components within these pathways varies among cell types and pathological conditions, reflecting differing metabolic requirements. Accurately measuring this dynamic system is essential for elucidating the metabolic distinctions between various cells and disease states.

By labeling specific carbons within the glucose molecule, the subsequent metabolic pathways of each segment can be determined. Uniformly labeled [U-^13^C]-glucose (with all carbons as ^13^C) and [1-^13^C]-glucose are commonly employed based on practicality and cost-effectiveness, respectively. Plasma samples are collected prior to tracer administration and at multiple intervals following the bolus injection. Glucose kinetics are subsequently evaluated by quantifying plasma tracer enrichment through MS or NMR spectroscopy and based on the calculation of the tracer-to-trace ratio for the target compound. In a recent study, an innovative methodology applied to hepatocytes was used, combining stable isotopes, such as [U-^13^C]-glucose, with recent analytical techniques [18]. The experimental protocol involved in vivo administration of labeled glucose to mice, enabling researchers to track the trajectory of ^13^C from systemic circulation into hepatic cells and their organelles. Using mass spectrometry, the incorporation of ^13^C into a wide range of metabolites present in hepatocytes was accurately quantified. Indirect calorimetry completed these measurements by assessing energy exchanges. This integrated approach allowed for detailed mapping of metabolic fluxes, revealed the cellular and subcellular architectures, and shed light on the adaptations of hepatocytes to physiological variations or experimental stimulation.

Stable isotopes of glucose are used for the investigation of tumor bioenergetics. The use of stable isotope tracers in human cancer research can provides insight into which metabolic pathways and nutrients are utilized within the tumor microenvironment, characterized by specific metabolic conditions and complex cell–cell interactions that are not fully reproduced in cell cultures [19,20,21]. A key aim of isotope tracing studies in cancer patients has been to identify the nutrients that support metabolic pathways in tumors and to compare these findings to adjacent tissues. The enhanced uptake and metabolism of glucose and subsequent conversion to fatty acid by fatty acid synthase (FAS) are commonly observed in cancer cells. The infusion of D-glucose during surgical resection of tumors, combined with subsequent ^13^C NMR spectroscopy, has shown elevated lactate production resulting from anaerobic glucose metabolism and a corresponding reduction in glucose-derived acetyl-CoA [22,23]. Stable isotopes, in combination with metabolomics, were used to study alterations in glucose metabolism in pancreatic cancer [24]. This approach can allow the flux of nutrients—particularly glucose—to be traced through different metabolic pathways, highlighting changes in monosaccharide production and commitment to glycosylation. Thanks to isotopic labeling and LC-MS analysis, it is possible to precisely quantify isotope incorporation into various metabolites, thus providing a detailed view of the metabolic adaptations in pancreatic cancer cells. This methodological coupling reveals how variations in glucose availability influence the biosynthesis of glycoconjugates and the dynamics of glycosylation pathways, offering key insights into tumor processes and paving the way for new targeted therapeutic strategies. While research has predominantly focused on glucose, other metabolites such as lactate, acetate, and glutamine have also been examined [25,26]. The oxidation of pyruvate derived from circulating [U-^13^C]-glucose has been identified in multiple tumor type analyses, including gliomas, brain metastases, breast tumors, non-small-cell lung cancer, clear cell renal cell carcinomas, and pediatric extracranial solid malignancies such as neuroblastomas and sarcomas [27,28,29,30,31,32].

The utilization of [1-^13^C]-glucose can enable the assessment of glucose pathway turnover, including glycolysis and the citric acid cycle, within different tissues including the human brain. In vivo studies have employed ^13^C NMR spectroscopy following ^13^C-glucose infusion into the forearm to non-invasively track 13C incorporation into various metabolites by collecting brain NMR spectra. These investigations have demonstrated that infusion of 1-^13^C-glucose induced ^13^C labeling at the C2, C3, and C4 positions of glutamine and glutamate, as well as at the C2 and C3 positions of aspartate in the human brain [33]. Multiple studies have identified metabolic alterations secondary to head trauma, such as increased lactate production and enhanced pentose phosphate pathway activity [34,35]. Glucose metabolism, in cases of head trauma, has also been investigated using microdialysis, an emerging minimally invasive technique involving the placement of a microdialysis catheter into brain parenchyma. This approach can allow 1-13C glucose to be directly infused into the brain, and subsequent samples of extracellular fluid can be taken to analyze glucose metabolic processes [36]. Stable isotopes were also used to trace the incorporation of ^13^C-glucose into nucleotide sugars and to observe differences in metabolic fluxes between induced pluripotent stem cells and fibroblasts, especially in disease models [37]. The use of stable isotopes coupled with metabolomics can enable a dynamic study of metabolic fluxes within cells. By labeling glucose, it has been possible to precisely track their incorporation into various metabolites and to map active metabolic pathways. This approach can provide detailed information on how cells redirect their metabolism in response to stimuli or under pathological conditions, thereby opening new perspectives for understanding cellular mechanisms and identifying potential therapeutic targets. The metabolomic analyses allow for precise quantification of biosynthetic pathway activity and identification of alterations associated with enzymatic deficiencies, such as those affecting phosphoglucomutase (PGM1).

The ^13^C-glucose breath test (^13^C-GBT) is a method proposed for assessing insulin sensitivity [38]. It is as a non-invasive alternative to the commonly used hyperinsulinemia euglycemic clamp, which is a more direct but invasive procedure. The ^13^C-GBT relies on cellular uptake of glucose, an insulin-dependent process, and thereby provides an overall measure of insulin sensitivity. When insulin is present, labeled ^13^C-glucose is taken up by muscular cells or other cells expressing GLUT4 through the translocation of GLUT4 to the cellular membrane, where it enters glycolysis, converts to acetyl-CoA, and generates ^13^CO_2_ that is subsequently exhaled in breath. In liver, labeled glucose and acetyl-CoA participates to glycogenogenesis and lipogenesis, two pathways stimulated by both glucose and insulin, where ^13^C is stored until labeled molecules are eventually oxidized. Exhaled ^13^CO_2_ is collected into glass tubes, where ^13^C enrichment can be measured by continuous flow MS. In cases of insulin resistance and type 2 diabetes, reduced glucose absorption can limit ^13^CO_2_ production. Research has indicated that the ^13^C breath test can provide insulin resistance measurements comparable to those obtained by more invasive clamp-based methods [39].

## 3. Protein and Amino Acid Metabolism

**Figure 2:**
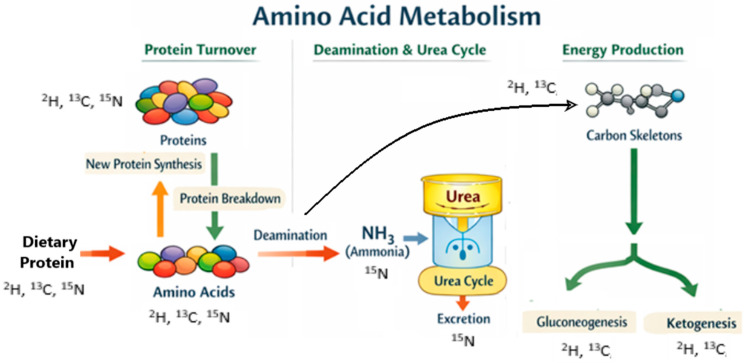
Protein and amino acid metabolism. The possible distribution of the stable isotopes ^2^H, ^13^C, and ^15^N in the different pools is indicated.

Stable isotope tracing techniques have emerged as valuable tools for evaluating fluctuations in protein and amino acid metabolism, offering insight not only at the level of the entire organism but also within specific tissues and individual proteins [40] (Table 3). Just as glucose and lipid metabolism shift in response to dietary intake, protein metabolism also varies throughout the feeding cycle—of which, 20 proteogenic amino acids are involved in protein turnover (Figure 2). To gain an accurate understanding of protein metabolism under different nutritional or pathological scenarios, it is important to distinguish between amino acids provided by the diet (exogenous) and those recycled during ongoing protein turnover (endogenous). Stable isotope-labeled amino acid tracers and stable isotope intrinsically labeled protein can achieve this detailed characterization in vivo in animals and humans.

When amino acids are catabolized, their amino groups are removed through deamination and ultimately excreted as urea in the urea cycle, while the remaining carbon skeleton is used for energy and carbon skeleton metabolic pathways (Figure 2). Amino acids fall into three categories based on their metabolic fate as glucogenic, ketogenic, or both. Glucogenic amino acids are converted into glucose via gluconeogenesis and ketogenic amino acids are broken down into acetoacetate or acetyl-CoA [17]. Early studies of protein metabolism involved administering ^15^N-labeled glycine, either by continuous or single-dose infusion, and then measuring ^15^N levels in urine and blood urea nitrogen [41]. This technique assumed that all metabolic nitrogen resided in a single pool, though this was later disproven [42]; as a result, continuous, primed infusions of ^13^C-leucine tracers have become the standard. Measuring ^13^CO_2_ in breath and blood ^13^C-leucine enrichment via mass spectrometry allows in vivo of leucine fluxes to be determined in order to assess whole-body protein metabolism [43]. Continuous, primed infusions of ^13^C-labled amino acid, particularly ^13^C-leucine tracers, and the assessment of plasma amino acid kinetics are the most widely used methods for determining whole-body protein synthesis, breakdown, and oxidation rates, as well as the net protein balance [43].

In the postabsorptive state—when no food has been recently consumed—plasma ^13^C-labled amino acid kinetics can be effectively measured by administering a constant infusion of one or more stable isotope-labeled amino acid tracers [43]. This continuous infusion enables the direct evaluation of how amino acids are produced, utilized, and recycled within the body under fasting conditions. However, in the postprandial state, following the ingestion of food, the situation becomes more complex due to the presence of exogenous amino acids derived from dietary protein. To precisely distinguish and quantify both endogenous amino acids released from body protein and exogenous amino acids entering the circulation from recently consumed food, it is necessary to combine the continuous tracer infusion approach with the ingestion of intrinsically labeled protein. By using protein sources that are themselves labeled with stable isotopes allows to accurately trace the appearance of dietary amino acids in the bloodstream, leading to a comprehensive assessment of amino acid turnover and protein metabolism in the fed state [43,44]. This combination of an amino acid tracer infusion with the ingestion of intrinsically labeled protein is the preferred method to quantify postprandial protein metabolism, as it is the only method to directly quantify exogenous plasma amino acid bioavailability and utilization.

Amino acid tracers are commonly administered intravenously, and the two principal approaches to assess tissue protein metabolism are arteriovenous balance and direct incorporation into protein methods [45]. The arteriovenous balance technique involves monitoring changes in tracer concentration throughout the body or across a limb. When applied correctly, this method can provide estimates of synthesis and degradation rates, as well as rates of intermediate metabolite formation, membrane transport, and substrate oxidation within specific tissues or limbs. For instance, tracking the rate at which a stable isotope tracer disappears from the arterial pool provides an estimate of synthesis, while its appearance in the venous pool indicates degradation. By comparing arterial and venous blood samples (known as arteriovenous [A–V] balance) from a particular tissue, such as a muscle group, it is possible to determine local protein turnover rates, though certain limitations should be considered [46]. In addition, once the tracer enters the intracellular amino acid pool, it binds to its respective acyl-tRNA and gets incorporated into tissue proteins. Tissue biopsies can then be used to chemically isolate and hydrolyze the target protein into its amino acids, allowing the tracer’s incorporation to be measured. By assessing tracer enrichment in the precursor pool—typically the amino-acyl-tRNA or, more conveniently, the intracellular amino acid pool—it is possible to calculate the fractional synthesis rate (FSR) of the protein [47,48].

The direct or precursor incorporation approach is commonly employed to measure the turnover of skeletal muscle protein or its fractional synthesis rate. As the body’s largest reservoir of protein, skeletal muscle serves as a crucial source of amino acids, especially during nutritional stress, when these building blocks are mobilized to support other organs. The balance between muscle protein synthesis (MPS) and muscle protein breakdown (MPB) determines changes in muscle mass. The use of stable isotope-labeled amino acid tracers can help characterize the intricate relationship between MPS and MPB, the mechanisms that support muscle maintenance and the consequences of imbalance [63,64]. These advanced tracing methods have revealed the continuous renewal of skeletal muscle proteins, which are constantly being built up and broken down [65]. One of the major regulators of this dynamic state is nutrient intake —specifically, the essential amino acids provided by meals—which serve as the primary driver for stimulating MPS together with anabolic hormones such as insulin [66,67,68].

D_2_O is a less invasive, cost-effective alternative to traditional labeled amino acid tracers for quantifying protein synthesis. While amino acid tracers allow short-term measurements (usually up to 12 h and require bedridden volunteers and intravenous cannulation), D_2_O enables long-term monitoring due to the slower water turnover. For example, in one study, participants received a D_2_O bolus and provided saliva samples over 8 days to track body water enrichment, along with muscle biopsies to assess deuterium incorporation. The results showed higher muscle protein synthesis rates in the exercised legs without significant hypertrophy [49]. In studies where a single dose of D_2_O was given to participants engaged in repeated unilateral resistance exercise over 8 days, D_2_O levels remained steadily elevated, allowing to detect differences in integrated anabolic responses in as little as 2 days [49]. Further research showed that during a period of continued resistance exercise for 6 weeks, early increases in D_2_O enrichment corresponded with increased muscle hypertrophy, suggesting that the most active period of muscle remodeling occurs early in a resistance training program [50]. Beyond its value for long-term MPS studies, D_2_O has also been directly compared with the traditional substrate-specific tracer, ring-[^13^C6]-phenylalanine, in shorter-term (3–6 h) measurements. When MPS was monitored for 3 hours after oral ingestion of 20 g of essential amino acids, both methods produced similar results for fractional synthesis rates and feeding responses [51]. Overall, D_2_O has proven to be a highly effective tool for capturing muscle protein metabolism over intervals ranging from just a few hours to several weeks or months.

Following periods without food (fasting), when MPB exceeds MPS and amino acids are released for use elsewhere in the body, nutrient intake triggers MPS and enables muscles to replenish their lost amino acids. Research has further explored how protein digestion speed affects whole-body protein metabolism after eating, often by using ^13^C-leucine infusions. These studies revealed that the rate of protein digestion can influence post-meal protein accumulation, pointing toward dietary strategies for preventing muscle loss, especially in aging populations. In younger adults, slowly digested proteins like casein have tended to promote greater net protein gain compared to rapidly digested proteins such as whey [52]. Conversely, in older adults, fast-digesting proteins were more effective at enhancing protein retention [53], highlighting their potential to counteract age-related declines in muscle mass. The MPS response following protein intake peaked at about 20–30 g of protein—roughly equal to 10–15 g of the essential amino acids—and was short-lived, lasting only 60 to 90 min before returning to baseline, even if amino acid levels in the blood remained high [54]. This phenomenon indicated that consuming more protein beyond this threshold would not further boost muscle growth; instead, the excess amino acids were directed toward oxidation and the production of urea [55].

Alongside the stimulation of MPS, the amino acids and carbohydrates from food also promote insulin secretion. Insulin acts to suppress muscle protein breakdown (MPB), thereby enhancing net protein gain [69,70]. These key anabolic signals govern the balance between MPS and MPB and help maintain skeletal muscle mass in healthy, physically active young adults. Notably, the extent of the anabolic response to nutrition is strongly influenced by muscle contraction. Stable isotopes methods have shown that performing resistance exercises before protein consumption prolongs the anabolic window, leading to higher rates of MPS—a boost that can last 24 to 48 h in those unaccustomed to training [57,58,59,60]. Regular resistance exercise accompanied by sufficient nutrition can result in extended periods where MPS exceeds MPB, ultimately driving muscle hypertrophy [49,50]. Conversely, inadequate muscle activity coud lead to a diminished MPS response to dietary protein, meaning that inactivity can contribute to muscle loss [61,62]. Stable isotope techniques were also used to examine the role of insulin in protein synthesis among individuals with type 1 diabetes [56]. By collecting blood samples and muscle biopsies before and after the infusion of [1-^13^C]-leucine and measuring leucine enrichment, it showed that [1-^13^C]-leucine incorporation into skeletal muscle proteins was unchanged or reduced during insulin infusion. Despite stable or increased plasma concentrations of most amino acids, the intramuscular concentrations of several amino acids decreased during insulin infusion. This may have limited any anabolic effect of insulin on protein synthesis. These results show no acute anabolic effect of systemic insulin infusion on skeletal muscle protein synthesis in insulin-withdrawn adult diabetic patients. It appears that reduced intracellular concentrations for a number of amino acids during insulin infusion, particularly tyrosine, may have constrained muscle protein synthesis.

## 4. Fatty Acids—Lipid and Lipoprotein Metabolism

Fatty acids consist of hydrocarbon chains of different sizes (short, medium, or long chain fatty acid). Stable isotopes are used to study lipid fatty acids metabolism, synthesiss and turnover [71] (Table 4).

**Figure 3:**
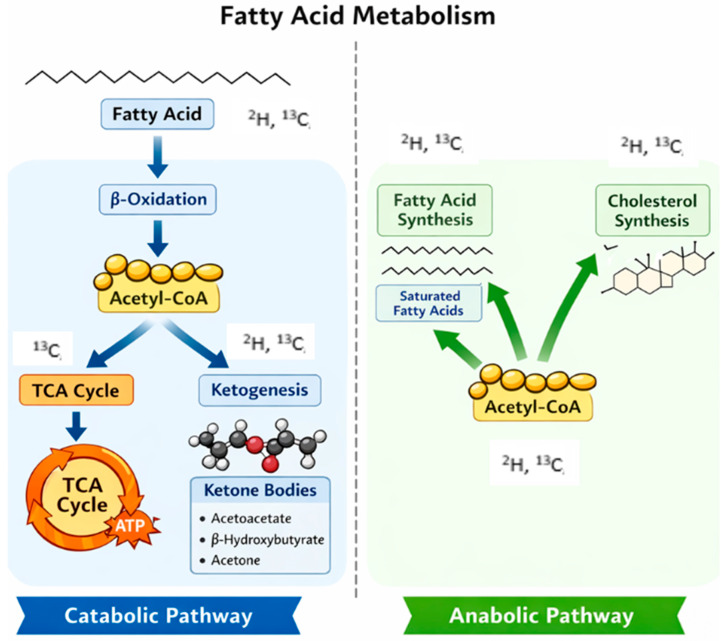
Fatty acid metabolic pathways. The possible distribution of the stable isotopes ^2^H and ^13^C in the different pools is indicated.

By catabolic processes in mammalian cells, fatty acid chains are progressively cleaved by β-oxidation into two-carbon units that combine with coenzyme A to form acetyl-CoA, which can then enter the tricarboxylic acid (TCA) cycle and ultimately produce ATP in the mitochondria (Figure 3). Alternatively, if the TCA cycle activity is low or is saturated, acetyl-CoA undergoes ketogenesis to form ketone bodies [17]. Through anabolic processes, saturated and monounsaturated fatty acids, as well as cholesterol, can be synthesized de novo from acetyl-CoA. In contrast, there is no de novo synthesis of polyunsaturated fatty acids with two or more double bonds. Furthermore, through anabolic processes, fatty acids can generate other biologically active molecules, such as signaling lipids, hormones, and other cell membranes components.

Fatty acid metabolism is investigated by administering stable isotope-labeled fatty acids—such as ^13^C- or ^2^H-labeled long-chain fatty acids—either intravenously or orally [72,73,74,75,76,77]. In addition, stable isotope-labeled acetyl-CoA precursors, including deuterated water (D_2_O), ^13^C-glucose, or ^13^C-labeled acetate and palmitate, are often used to track the synthesis of glyceridic fatty acids and cholesterol. Palmitate is a preferred tracer, particularly for measuring non-esterified fatty acid (NEFA) production. De novo lipogenesis is monitored by observing how ^13^C- or D_2_O-derived acetyl-CoA units are incorporated into glyceridic fatty acids, which increases their mass isotopomer abundance compared to naturally occurring levels. The distribution of these isotopomers can reflect the rate of fatty acid synthesis, as it depends on the degree of label incorporation. Lipogenic flux rates are then determined by analyzing these enrichment patterns using mass isotopomer distribution analysis (MIDA) [78,79,80,81].

Stable isotope techniques can track how lipids are metabolized in the body, especially when using multiple tracers together—such as those that reveal ketogenic and citrate synthase activity in the liver. When paired with measurements of ^13^CO_2_ in breath and indirect calorimetry, these methods can estimate both the oxidative and nonoxidative metabolic rates of dietary or circulating fatty acids. By combining these data with arteriovenous blood sampling and tissue biopsies, fatty acid uptake and release can be determined in white adipose tissue as well as the muscle, heart, and liver, providing insight into how the body utilizes dietary fats. These approaches have greatly advanced our understanding of de novo lipogenesis in response to diet and disease, although certain limitations of the acetyl-CoA labeling strategy are discussed [104]. For metabolic dysfunction-associated steatotic liver disease (MASLD), formerly known as non-alcoholic fatty liver disease (NAFLD), breath tests using ^13^C-labeled substrates—like α-ketoisocaproic acid, octanoic acid, or some other substrates—offer an indirect, cost-effective, and simple way to measure metabolic dynamic liver function. These tests help assess mitochondrial oxidation, which can be compromised early in NAFLD [82]. Additionally, other stable isotope-based methods can be used to study mitochondrial dysfunction, oxidative stress, and the development of fibrosis in NAFLD [83].

The use of [U-^13^C]-palmitate can enable the fate of NEFAs in human skeletal muscle to be tracked, particularly in relation to muscle activity. During studies, [U-^13^C]-palmitate was infused into a forearm vein over 5 hours as participants performed single-leg knee extension exercises. Blood samples taken at intervals were used to assess the plasma enrichment of ^13^C-palmitate in NEFAs. The findings revealed that muscle contractions increased NEFA oxidation, reducing their storage in muscle tissue and demonstrating that exercise shifted the balance towards using NEFAs for energy [84]. When studying phospholipids, the use of stable isotope-labeled fatty acids only revealed the origin of phospholipid species that contained the labeled fatty acid or its broken down products. Alternatively, isotopically labeled phospholipid headgroups, such as methyl-^2^H_2_-choline chloride, 4-^2^H-ethanolamine, 6-^2^H-myo-inositol, and 3-^2^H-serine, can be used to trace enrichment and, in certain cases, metabolic flux rates—these methods have been well-documented [85,86]. The lowest enrichment levels typically detectable with these approaches were around 0.05–0.1%, though some important limitations have remained [105,106]. These methods contributed to a better understanding of the role of glycerophospholipids have, which compose the main lipid category of mammalian cell membranes. In addition to their function as building blocks of the lipid bilayer, these lipids play a vital role in cellular functions, including the regulation of transport processes, protein function and signal transduction and are also essential components of lipoproteins and influence their function and metabolism.

D_2_O is also widely used to measure lipid turnover, providing insight into their tissue-specific metabolic pathways. It can effectively track de novo lipogenesis and cholesterol synthesis in vivo [87,88,89]. Incorporating deuterium into plasma triglycerides can help determine the fractional synthesis rate of fatty acids and cholesterol. Research using D_2_O has shown that high-carbohydrate diets increased de novo lipogenesis [90], while insulin receptor mutations or inhibitory antibodies were linked to low triglyceride levels and normal high-density lipoproteins (HDL) cholesterol. Mutations in AKT2 (RAC-beta serine/threonine-protein kinase), which were related to post-receptor signaling, caused elevated de novo lipogenesis, increased hepatic fat, and reduced HDL cholesterol, highlighting the significance of post-receptor hepatic insulin resistance in metabolic dyslipidemia and hepatic steatosis [91,92].

The ^13^C-octanoic acid breath test offers a convenient, non-invasive approach for assessing gastric emptying [94]. In this procedure, a patient consumed a meal containing 13C-labeled octanoic acid bound to egg yolk. Once ingested, the octanoic acid was released as the food was mixed and churned in the stomach. It then quickly entered the duodenum and was transported to the liver via the portal vein. As a medium-chain fatty acid, ^13^C-octanoic acid was taken up by cells and directed into the mitochondria, where it underwent β-oxidation, producing two-carbon acetyl-CoA units. These units entered the TCA cycle and were ultimately metabolized to ^13^CO_2_, which appeared almost immediately in the patient’s exhaled breath. As for other breath tests, the patient provided exhaled breath samples, which were collected in sealed glass tubes and then analyzed for ^13^C enrichment using continuous flow MS. The resulting data were modelled to quantify gastric emptying rates [93,94,95]. This breath test is safe, affordable, and efficient. Studies have shown that patients with diabetes often have delayed gastric emptying [96,97]. Similarly, individuals with amyotrophic lateral sclerosis (ALS) exhibited delayed gastric emptying of solids, suggesting that ALS affected more than just the motor neurons, highlighting its multisystem nature [98].

Efficient transport of triglycerides between the intestine, adipose tissue, liver, and muscle is precisely regulated to ensure an optimal use of these nutrients. Triglycerides are vital for energy, so their transport is tightly managed to match the body’s needs; however, when these needs are exceeded, lipoproteins can deposit fatty acids in adipose tissue, which can lead to weight gain and obesity. Triglyceride-rich lipoproteins and their cholesterol-rich remnants play a significant role in the development of atherosclerosis [99]. The application of stable isotope tracers and advanced modelling techniques has greatly enhanced our understanding of the mechanisms driving lipid disorders in individuals at increased risk for cardiovascular disease [100].

The intestine and liver release chylomicrons and very low-density lipoproteins (VLDL) into the bloodstream, both of which are rich in triglycerides. These particles are built with apolipoprotein B (apoB), which is in two forms, apoB-100 and apoB-48 [101,102]. These apo-lipoproteins are then recognized in target adipose tissue which allow the deposition of triglycerides for storage. ApoB-100, a very large protein of 4536 amino acid residues, is produced in the liver and is crucial for very low-density lipoproteins (VLDL) assembly. ApoB-48 is produced in the intestine and is needed for chylomicron formation. According to the transport and content of triglycerides different particle forms of lipoproteins are produced including intermediate-density lipoproteins (IDL), low-density lipoproteins (LDL), and various remnants. These cholesterol-rich particles, along with LDL, are considered major contributors to arterial plaque formation. Genetic studies have drawn connections between high plasma triglyceride levels and increased risk of cardiovascular disease and have pointed specific proteins that regulate triglyceride movement in the body. Stable isotope tracers can be used to label proteins in different lipoprotein classes, such as APOB-100 in VLDL, IDL, and LDL; APOB-48 in chylomicrons and remnants; and apo(a) in lipoprotein(a). This makes it possible to track how a particular protein moves through multiple lipoprotein groups. Kinetic studies in humans, using stable isotope tracers, have been key to uncovering the complexity of triglyceride- and cholesterol-rich lipoprotein metabolism [99]. The use of stable isotopes allow how disruptions—whether from diet or genetic changes—can raise the risk for conditions like atherosclerosis and pancreatitis to be investigated. Novel drugs are being developed to target specific molecules in these regulatory pathways, offering new opportunities for prevention and treatment of lipid-related diseases.

In contrast to VLDL, and their role in the development of atherosclerosis, HDL are believed to play a protective role through reverse cholesterol transport—a mechanism that enables the removal of excess cholesterol from peripheral tissues. This process is controlled by apolipoprotein A-I, the primary structural protein of HDL, as well as other HDL-associated proteins. Interestingly, interventions that elevate HDL cholesterol have not consistently reduced cardiovascular events, suggesting that the current understanding of HDL biology remains incomplete. Stable isotope kinetic studies offer the ability to label proteins originating from different organs, such as HDL synthesized in the liver or small intestine [103]. This allows to monitor how specific interventions affect both hepatic and intestinal HDL production. In practice, in vivo protein labeling involves administering a stable isotope-labeled amino acid—such as deuterium ^2^H- or ^13^C-labeled leucine or phenylalanine—which is then incorporated into newly synthesized proteins, including apoprotein A1 (APOA1) produced by the liver or intestine, and subsequently secreted into the plasma. These tracer-based studies are invaluable for probing the dynamics of HDL-mediated reverse cholesterol transport and for understanding HDL metabolism as it relates to cardiovascular disease. Using stable isotopes can allow HDL metabolism to be tracked in vivo in the human body [103]. It appears that HDL particles are highly heterogeneous, with varying protein compositions and kinetic properties that likely influence their function and capacity for cholesterol removal.

## 5. Monitoring Daily Energy Expenditure

Total daily energy expenditure is a critical variable in human health and physiology, that represents a strong risk factor for many diseases. It is measured in free-living conditions by the indirect calorimetry doubly labeled water method [1,7,8]. This is a strictly non-invasive method in which individuals drink a dose of water doubly labeled with the stable isotopes of ^2^H and ^18^O. Isotope enrichment is measured 3–6 h after dosing and over the following 1–2 weeks while individuals engage in their habitual activities and subsequently, the elimination rate of each isotope can be measured. As such, ^2^H is eliminated only as ^2^H_2_O in urine and other body fluids, whereas ^18^O is eliminated as H_2_^18^O and C^18^O_2_ in exhaled air. Consequently, ^18^O has a higher elimination rate than ^2^H, and the difference in the elimination rates of the two isotopes is used to calculate the VCO_2_ over the entire period of measurements and to calculate energy expenditure from VCO_2_.

The International Atomic Energy Agency (IAEA) has established a doubly labeled water method database on the effects of age on human energy expenditure [107]. The database includes free-living total daily energy expenditure data from thousands of individuals aged 8 days to 96 years from various countries. Free-living total daily energy expenditure was measured by doubly labeled water and many of the tested individuals also had their resting energy expenditure measured by indirect calorimetry. Fat-free mass was calculated using the dilution spaces of ^2^H and ^18^O along with hydration constants. Fat mass was derived as the difference between body weight and fat-free mass.

In this large IAEA database, fat-free mass and fat mass have been shown to display large variations throughout the life course [8,108]. On average, fat-free mass increased progressively with age and reached its maximum value at 30 years in men and women [108]. Fat mass also increased progressively with age but reached its maximum value later in life, at approximately 50 years of age [108]. Since fat-free mass and fat mass are the major determinants of resting energy expenditure, it follows their life course trajectories. Fat-free mass and fat mass explained at least 53% of the variance in resting energy expenditure within different age ranges (1–20 years, 20–60 years, and 60 or more years) [8]. Resting energy expenditure also varies over the life course independently of fat-free mass and fat mass. Statistically adjusting resting energy expenditure for differences in fat-free mass and fat mass can allow the detection of changes in resting energy expenditure throughout life, independent of the changes in body weight and body composition.

The IAEA database was used together with some additional sources of data to determine the effects of age on resting energy expenditure, adjusted for fat-free mass and fat mass, in over 2000 individuals [8]. The trajectory of total daily energy expenditure was analyzed over the life course from these diverse databases of total expenditure measured by the doubly labeled water method for males and females in free-living conditions. Resting energy expenditure was expressed as a percentage of the value predicted based on fat-free mass and fat mass using a linear regression model derived from individuals aged 20 to 60 years. The analysis showed that total expenditure increased with fat-free mass in a power–law manner, with four distinct life stages. Fat-free mass-adjusted expenditure accelerated rapidly in neonates to ~50% above adult values at ~1 year; declined slowly to adult levels by ~20 years; remained stable in adulthood (20 to 60 years), even during pregnancy; and then declined in older adults. As concluded by the authors, “these changes shed light on human development and aging and should help shape nutrition and health strategies across the life span”.

## 6. Conclusions and Perspectives

Over several past decades, substrate-specific stable isotope tracers have greatly advanced our understanding of glucose, fatty acid, and amino acid metabolism in health and disease. Stable isotope-based methods have profoundly transformed the study of energetic nutrient metabolic fluxes in humans, enabling precise characterization of processes at the systemic, tissue, organ and cellular levels, both under physiological and pathological conditions (Table 5).

While stable isotope-based methods have revolutionized metabolic research, they are not without limitations. These include technical and methodological constraints; sample and invasiveness issues; complexity in data interpretation, individual variability; challenges in integrating new technologies; and cost/accessibility concerns. In the coming years, ongoing technological advances and methodological refinements are expected to address many of these limitations. These technical limitations include the need for highly specialized analytical equipment (such as mass spectrometry and NMR), which may not be widely available in all research or clinical settings. The precision of isotope enrichment detection is currently limited by the sensitivity of available technologies, although ongoing advances are expected to improve this. Another limitation is that some stable isotope techniques require relatively large sample volumes or invasive procedures (e.g., tissue biopsies for protein synthesis studies), which can limit their application, especially in vulnerable populations or for long-term monitoring. While less invasive methods (like using D_2_O and sampling saliva, urine, or breath) are being developed, these are not yet universally applicable for all types of metabolic studies. The interpretation of metabolic flux data from stable isotope studies can be complex, requiring advanced modelling and a deep understanding of metabolic networks. This complexity can introduce uncertainty or variability in results, especially when comparing across different studies or populations. Responses can vary significantly between individuals due to genetic, environmental, and lifestyle factors. This individual variability can limit the generalizability of findings and underscores the need for personalized approaches. Although the integration of stable isotope methods with artificial intelligence, omics, and miniaturized detection tools is promising, these approaches are still in development. Their full potential and limitations are not yet fully realized, and further research is needed to validate and standardize these combined methodologies. The cost of stable isotope tracers and the associated analytical procedures can be high, potentially limiting access for some research groups or clinical applications.

There are promising areas for potential developments. Future advances in analytical technology, miniaturization, integration with AI and omics, non-invasive sampling, and methodological refinement are expected to address several of the current limitations of stable isotope-based metabolic studies. These developments will enhance precision, accessibility, and personalization; support a deeper understanding of human metabolism; and drive progress in global health and personalized medicine. Advances in analytical technologies (such as mass spectrometry and NMR) will improve the sensitivity and precision of isotope enrichment detection. This will allow for more accurate measurements, even with smaller sample volumes. The development of miniaturized and portable detection devices will make stable isotope analysis more accessible, enabling less invasive sampling (e.g., saliva, urine, breath) and broader application in clinical and research settings. Ongoing research is expanding the use of stable isotopes in non-invasive methods, such as breath, saliva, and urine tests. This will reduce the need for large sample volumes and invasive procedures like tissue biopsies, making studies safer and more feasible for vulnerable populations and long-term monitoring.

The integration of stable isotope approaches with omics technologies (metabolomics, proteomics) will help manage the complexity of metabolic flux data. AI can assist in advanced modelling, interpretation, and standardization, reducing uncertainty and variability in results. Combining isotopic methods with omics and imaging will provide a more systemic and integrated view of metabolism, supporting personalized medicine and translational research. This will help tailor nutritional and therapeutic strategies to individual metabolic profiles. Advances in technology and methodology will allow researchers to better account for genetic, environmental, and lifestyle differences among individuals. This will improve the generalizability of findings and support the development of personalized diagnostic and therapeutic interventions. As detection technologies become more efficient and miniaturized, the cost of stable isotope tracers and analytical procedures is expected to decrease. This will make these methods more accessible to a wider range of research groups and clinical applications. Ongoing research will focus on validating and standardizing new combined methodologies (e.g., isotope tracing with AI and omics), ensuring their reliability and reproducibility across different studies and populations.

These methods can allow precise measurement of metabolic markers and pathways, revealing insights into conditions like diabetes and liver disease. By using tracers such as ^2^H, ^13^C, ^15^N, and ^18^O, it is now possible to track the fate of glucose, fatty acids, and amino acids, and to identify metabolic alterations associated with diseases such as diabetes, liver disease, or cancer. The integration of advanced analytical technologies, like mass spectrometry and NMR, has enabled fine and dynamic analysis of metabolic pathways, paving the way for increased understanding of pathophysiological mechanisms and targeted evaluation of therapeutic interventions. Ongoing advances in technology will further enhance the precision of isotope enrichment, enable detection with smaller samples, and allow the development of non-invasive methods through the detection of isotope enrichment in saliva, urine, or breath, thereby deepening our understanding of human metabolism. Furthermore, as the use of nuclear techniques becomes more widespread, their integration with new tools, such as artificial intelligence and new analytical methods, will greatly enhance our ability to understand and assess metabolic diseases. On one hand, the ongoing evolution of isotope enrichment techniques and the miniaturization of detection tools will allow for less invasive analyses, using smaller volumes. On the other hand, combining isotopic approaches with artificial intelligence, imaging methods, and omics methods (metabolomics, proteomics) will offer a systemic and integrated view of human metabolism, fostering the identification of new biomarkers and therapeutic targets. Finally, expanding these methods to personalized medicine and translational research will help to better understand individual variations and to tailor nutritional or medical strategies according to each patient’s metabolic profile. Thus, stable isotopes are emerging as essential tools to meet the challenges of contemporary biomedical research and to improve human health on a global scale.

## Figures and Tables

**Table 1:** Stable isotope-based methods to study energy nutrient metabolic fluxes [1,2,3].

Field	Description	Examples/Applications
Regulation of metabolic fluxes	Results from the balance between absorption, synthesis, transformation, catabolism, and excretion of nutrients and the dynamic interactions within complex networks of biomolecules.	Metabolism of carbohydrates, lipids, proteins, and their precursors (glucose, fatty acids, amino acids).
Use of stable isotopes	Metabolic fluxes of energy nutrients in vivo at different levels (systemic, tissue, organ) in healthy or diseased humans.	Analysis of metabolic alterations in various pathologies and comparison of healthy/diseased states.
Advanced analytical techniques	High-precision NMR spectroscopy and mass spectrometry (MS) mainly using stable isotopes.	Measurement of metabolic fluxes and characterization of metabolic pathways.
Stable isotopes used	^2^H, ^13^C, ^15^N, ^18^O differ from the most abundant natural isotopes by the number of neutrons.	Labeling of precursors: glucose (^2^H, ^13^C), fatty acids (^2^H, ^13^C), and amino acids (^2^H, ^13^C, ^15^N, ^18^O).
D_2_O (heavy water)	Versatile tracer allows simultaneous labeling of several substrates, rapid diffusion in body water, and integration into multiple metabolic pathways.	Prolonged measurements over several days/weeks/months of carbohydrate, lipid, and protein metabolism.
Nuclear techniques/imaging	Complement classical biochemical methods, allowing precise and safe evaluation of numerous metabolic parameters.	Determination of body composition, energy expenditure, nutrient bioavailability, storage, and metabolism.

**Table 2:** Stable isotope-based methods for glucose metabolic pathways.

Topic	Description	Methods and Techniques	Applications	Findings and Outcomes	References
Glucose as Energy Source	Principal energy source for cellular processes and supplies ATP and carbon substrates.	-	Brain, erythrocytes, and fast-twitch skeletal muscle, heart (with fatty acids and amino acids).	Critical for maintenance, growth, and proliferation.	-
Glucose Metabolism Pathways, Cellular Energy Production, and Biosynthesis	Multiple pathways and metabolic routes of carbon flux.		Glycolysis, pentose phosphate pathway, glycogen metabolism, TCA cycle, and fatty acid and amino acid metabolism.	Glucose converted to pyruvate, lactate, acetyl-CoA, and citrate; directed into PPP or stored as glycogen.	[17]
Measurement of Glucose Metabolism	Expression of enzymatic components varies among cell types and conditions.	Glucose tracers, labeled glucose (U-^13^C-glucose, 1-^13^C-glucose), MS, and NMR spectroscopy.	Assess metabolic fate of glucose and distinguish between cells and disease states.	Tracer-to-trace ratio calculated for target compound.	[12]
Innovative Methodology in Hepatocytes	In vivo administration of labeled glucose.	Stable isotopes ([U-^13^C] glucose), mass spectrometry, and indirect calorimetry.	Track ^13^C from circulation into hepatic cells and organelles.	Detailed mapping of metabolic fluxes, cellular/subcellular architecture, and adaptation insight.	[18]
Stable Isotopes and Tumor Bioenergetics	Investigation of tumor metabolism using stable isotope tracers.	Infusion of D-glucose, ^13^C NMR spectroscopy, LC-MS, and metabolomics.	Study metabolic pathways and nutrients in tumors, compared to adjacent tissues.	Elevated lactate production, reduced glucose-derived acetyl-CoA, and changes in glycosylation.	[19,20,21,22,23,24]
Other Metabolites Studied	Besides glucose, examination of metabolites like lactate, acetate, and glutamine.	Oxidation analysis using [U-^13^C]-glucose.	Multiple tumor types: gliomas, brain metastases, breast tumors, NSCLC, ccRCCs, neuroblastoma, and sarcoma.	Pyruvate oxidation from [U-13C]-glucose identification.	[25,26,27,28,29,30,31,32]
13C-Glucose in Brain Studies	Assessment of glucose pathway turnover in brain.	^13^C NMR spectroscopy, microdialysis, and infusion of 1-^13^C glucose.	Non-invasive tracking of 13C incorporation and study of metabolic alterations after head trauma.	13C labeling of glutamine, glutamate, and aspartate; increased lactate and enhanced PPP activity.	[33,34,35,36]
Stable Isotopes in Stem Cells and Fibroblasts	Tracing ^13^C-glucose into nucleotide sugars and metabolic fluxes in hiPSC and fibroblasts.	Stable isotopes, metabolomics, and labeling glucose.	Study metabolic responses to stimuli or pathology.	Precise tracking of metabolite incorporation, mapping of active pathways, and identification of enzymatic deficiencies.	[37]
^13^C-Glucose Breath Test (13C-GBT)	Non-invasive assessment of insulin sensitivity.	^13^C-GBT, breath collection, and continuous flow MS.	Alternative to hyperinsulinemia euglycemic clamp.	Measures insulin sensitivity via ^13^CO_2_ production, comparable to clamp-based methods	[38,39]

**Table 3:** Stable isotopes-based methods for protein and amino acid metabolic pathways.

Technique and Method	Description	Application	Advantages	Limitations	Key Findings and Notes	References
Stable isotope tracing	Use of stable isotope-labeled amino acid tracers.	Evaluating protein and amino acid metabolism in vivo.	Distinguishes exogenous vs. endogenous amino acids.	Requires specialized tracers and analysis	Allows detailed characterization in animal and human	[40]
^15^N-labeled glycine infusion	Administering ^15^N-glycine, then measuring ^15^N in urine and blood urea.	Early studies of protein metabolism.	Simple measurement of metabolic nitrogen.	Assumed single nitrogen pool, later disproven.	Basis for later tracer methods.	[41,42]
^13^C-leucine tracer infusion	Continuous, primed infusions of ^13^C-leucine.	Determining in vivo leucine fluxes.	Standard for protein metabolism studies.	Requires mass spectrometry.	Measures ^13^CO_2_ in breath and blood.	[43]
Plasma amino acid kinetics	Assessment via stable isotope-labeled amino acid tracers.	Whole-body protein synthesis, breakdown, and oxidation rates.	Direct evaluation in postabsorptive state.	Complexity increases postprandially.	Requires continuous infusion and/or labeled protein ingestion.	[43,44]
Arteriovenous balance	Monitoring tracer concentration across limb/tissue.	Tissue protein metabolism estimation.	Estimates synthesis, degradation, transport, and oxidation.	Requires arterial and venous sampling.	Enables local protein turnover rates.	[45,46]
Direct incorporation method	Tracer binds to acyl-tRNA, then incorporates into proteins.	Fractional synthesis rate (FSR) calculation.	Measures protein-specific synthesis.	Requires tissue biopsies.	Assesses precursor pool enrichment.	[47,48]
D_2_O (deuterium oxide) tracing	Bolus of D_2_O and saliva/muscle samples for deuterium incorporation.	Long-term muscle protein synthesis monitoring.	Less invasive, cost-effective, and allows long-term study.	Short-term resolution lower than amino acid tracers.	Captures muscle protein metabolism over hours to months.	[49,50,51]
Protein digestion speed studies	^13^C-leucine infusion to measure protein accumulation post-meal.	Effect of digestion speed on whole-body protein metabolism.	Reveals dietary strategies for muscle maintenance.	Short-lived MPS response.	Slow-digesting proteins favor net gain in young, while faster in older adults.	[52,53,54,55]
Insulin infusion with stable isotopes	[1-^13^C]-leucine infusion with blood/muscle samples pre/post insulin.	Role of insulin in protein synthesis (type 1 diabetes).	Examines anabolic signals and MPB suppression.	Requires biopsies and infusions.	Insulin reduces [1-^13^C]-leucine incorporation into muscle proteins.	[56]
Exercise and protein feeding studies	Stable isotope tracers before/after resistance exercise and protein intake.	Effect of exercise on MPS and anabolic window.	Shows exercise prolongs MPS response to protein.	Requires multiple samples and interventions.	Anabolic window lasts 24–48 h post-exercise in untrained.	[57,58,59,60]
Muscle inactivity studies	Stable isotope methods to assess MPS response to inactivity.	Impact of inactivity on muscle protein metabolism.	Highlights risk of muscle loss due to inactivity.	Not specified.	Inactivity leads to diminished MPS response to protein.	[61,62]

**Table 4:** Stable isotopes-based methods for lipid and lipoprotein metabolic pathways.

Topic	Key Details	Stable Isotope Technique	Metabolites and Tracers	Application and Measurement	References
Fatty Acid Metabolism	Hydrocarbon chains, β-oxidation, acetyl-CoA, TCA cycle, and ketogenesis.	Stable isotope-labeled fatty acids.	^13^C- and ^2^H-labeled long-chain fatty acids.	Metabolism and synthesis, turnover	[71]
De Novo Lipogenesis	Acetyl-CoA incorporation and mass isotopomer abundance.	Stable isotope-labeled acetyl-CoA precursors.	D_2_O, ^13^C-glucose, and ^13^C-acetate and palmitate.	Synthesis of glyceridic fatty acids and cholesterol.	[72,73,74,75,76,77]
Lipogenic Flux Rates	Mass isotopomer distribution analysis (MIDA).	Stable isotope enrichment analysis.	^13^C and D_2_O.	Rate of fatty acid synthesis.	[78,79,80,81]
Metabolic Dysfunction-Associated Steatotic Liver Disease (MASLD/NAFLD)	Indirect, cost-effective liver function measurement.	Breath tests with stable isotope-labeled substrates.	^13^C-α-ketoisocaproic acid and ^13^C-octanoic acid.	Mitochondrial oxidation assessment.	[82]
Mitochondrial Dysfunction & Oxidative Stress in NAFLD	Study of fibrosis development.	Stable isotope-based methods.	Not specified.	Mitochondrial dysfunction and oxidative stress.	[83]
NEFA Fate in Muscle	Muscle contraction increases NEFA oxidation.	[U-^13^C]-palmitate infusion.	[U-^13^C]-palmitate.	NEFA oxidation during exercise.	[84]
Phospholipid Metabolism	Origin and flux of phospholipid species.	Stable isotope-labeled fatty acids and headgroups.	Methyl-^2^H_2_-choline chloride, 4-^2^H-ethanolamine, 6-^2^H-myo-inositol, and 3-^2^H-serine.	Phospholipid enrichment and flux rates.	[85,86]
Lipid Turnover & Synthesis	De novo lipogenesis, cholesterol synthesis.	D_2_O incorporation.	D_2_O.	Fractional synthesis rate of fatty acids and cholesterol.	[87,88,89,90]
Insulin Resistance & Lipogenesis	AKT2 mutations, hepatic fat, and HDL cholesterol.	D_2_O tracing.	D_2_O.	De novo lipogenesis, metabolic dyslipidemia.	[91,92]
Gastric Emptying	Non-invasive assessment of diabetes and ALS.	^13^C-octanoic acid breath test.	^13^C-octanoic acid bound to egg yolk.	Gastric emptying rates.	[93,94,95,96,97,98]
Triglyceride Transport	Intestine, adipose tissue, liver, and muscle.	Stable isotope tracers.	Not specified.	Mechanisms of lipid disorders and atherosclerosis risk.	[99,100]
Lipoprotein Particles	Chylomicrons, VLDL, IDL, LDL, and remnants.	Stable isotope protein labeling.	APOB-100, APOB-48, and apo(a).	Tracking protein movement in lipoprotein groups.	[99,101,102]
HDL Metabolism	Reverse cholesterol transport, protein heterogeneity.	Stable isotope kinetic studies.	Deuterium ^2^H- and ^13^C-labeled leucine or phenylalanine.	HDL synthesis, function, and cholesterol removal.	[103]

**Table 5:** Conclusion and perspectives on stable isotope-based methods.

Aspect	Description
Isotope Tracers Used	^2^H, ^13^C, ^15^N, and ^18^O.
Stable Isotope Tracers for Metabolic Flux Studies Measurement	Advanced understanding of glucose, fatty acid, and amino acid metabolism in health and disease; enabled precise characterization at systemic, tissue, and organ levels under physiological and pathological conditions; precise measurement of metabolic markers and pathways; and insights into diabetes and liver disease.
Analysis Type, Technological Advances, and Prospects	Fine and dynamic analysis of metabolic pathways of enhanced precision of isotope enrichment, detection with smaller samples, and non-invasive methods via saliva, urine, and breath.
Integration Approaches with New Tools and Analytical Technologies	Mass spectrometry, NMR, and isotopic approaches with artificial intelligence, imaging methods, omics (metabolomics, proteomics), and nuclear techniques to enhance the ability to assess metabolic diseases.
Global Health Impact	Stable isotopes as essential tools to improve human health globally.
Strength and Benefits	Systemic and integrated view of metabolism; and identification of new biomarkers and therapeutic targets.
Personalized Medicine	Expansion to personalized medicine and translational research; and tailored strategies to metabolic profile.

## Data Availability

No new data were created or analyzed in this study.

## List of Abbreviations

APOA1	Apoprotein A1
apoB	Apolipoprotein B
AI	Artificial Intelligence
FSR	Fractional Synthesis Rate
HDL	High-Density Lipoproteins
IDL	Intermediate-Density Lipoproteins
LDL	Low-Density Lipoproteins
NAFLD	Non-Alcoholic Fatty Liver Disease
NEFA	Non-Esterified Fatty Acid
NMR	Nuclear Magnetic Resonance
MIDA	Mass Isotopomer Distribution Analysis
MS	Mass Spectrometry
MPS	Muscle Protein Synthesis
AKT2	RAC-Beta Serine/Threonine-Protein Kinase
TCA	Tricarboxylic Acid
^13^C-GBT	^13^C-Glucose Breath Test
VLDL	Very Low-Density Lipoproteins
